# Play dough as an educational tool for visualization of complicated cerebral aneurysm anatomy

**DOI:** 10.1186/1472-6920-5-15

**Published:** 2005-05-10

**Authors:** Behzad Eftekhar, Mohammad Ghodsi, Ebrahim Ketabchi, Arman Rakan Ghazvini

**Affiliations:** 1 Department of Neurosurgery, Sina Hospital, Tehran University, Iran

## Abstract

**Background:**

Imagination of the three-dimensional (3D) structure of cerebral vascular lesions using two-dimensional (2D) angiograms is one of the skills that neurosurgical residents should achieve during their training. Although ongoing progress in computer software and digital imaging systems has facilitated viewing and interpretation of cerebral angiograms enormously, these facilities are not always available.

**Methods:**

We have presented the use of play dough as an adjunct to the teaching armamentarium for training in visualization of cerebral aneurysms in some cases.

**Results:**

The advantages of play dough are low cost, availability and simplicity of use, being more efficient and realistic in training the less experienced resident in comparison with the simple drawings and even angiographic views from different angles without the need for computers and similar equipment. The disadvantages include the psychological resistance of residents to the use of something in surgical training that usually is considered to be a toy, and not being as clean as drawings or computerized images.

**Conclusion:**

Although technology and computerized software using the patients' own imaging data seems likely to become more advanced in the future, use of play dough in some complicated cerebral aneurysm cases may be helpful in 3D reconstruction of the real situation.

## Background

Imagination of the three-dimensional (3D) structure of cerebral vascular lesions using two-dimensional (2D) angiograms is one of the skills that neurosurgical residents should achieve during their training. Although ongoing progress in computer software and digital imaging systems has facilitated viewing and interpretation of cerebral angiograms significantly, the 2D nature of these images makes them still far from ideal. These facilities are not always readily available in many situations. Much of the actual mastering is achieved experientially through work on cadavers or in operating theatres.

Three-dimensional models made from materials such as wax, bronze and ivory have been used in the teaching of medicine for many centuries.

It is thought that the first three-dimensional model of the vascular tree was created by a follower of Mondino de'Luzzi in the 14th century. Molten wax was injected into the vascular system, forming a cast that was carefully dissected out from the surrounding tissue [1,2]. In the 17th and 18th centuries artists such as Ercole Lelli (1702–1766) turned to colored waxes to realistically recreate dissected figures and organs[3,4]. More recently, technology has started to displace this traditional way of teaching with the development of high quality visual and often interactive three-dimensional (3D) computer-generated images [5]. Computerized 3D models have not only been used for teaching anatomy and pathology, but also in different fields of neurosurgical training and interpretation of neuroradiological images [6-8].

Although there are some anatomic variations among different patients in all organs, the individual anatomy of brain vascular lesions in particular needs to be studied precisely before surgical intervention. This study is traditionally done through 2D angiograms. While modalities like Computed Tomography (CT) angiograms and Magnetic Resonance Angiography (MRA) gather data three dimensionally, this is only available through 2D monitors or printed materials. Current commercial computer software have made manipulation and viewing of the Magnetic Resonance Imaging (MRI) or CT images much easier, but their capabilities are far from ideal.

An evolving technology with potential application to medicine is three-dimensional printing, also known as rapid prototyping. Devices "print" three-dimensional data sets into solid models using various materials such as plastic, wax, and metal. Radiographic studies containing volumetric data can therefore be made into realistic three-dimensional physical anatomic models[2].

It seems that the trend is towards more complicated imaging technologies, both in training and surgical intervention.

Play dough is an old toy that has been used both in play and learning by our children for a long time. It is a time-honored educational tool on account of its simplicity of use, its pliability and relative inexpensiveness [9]. We could not find any reference in medical literature regarding similar usage as an educational tool for neurosurgical training in visualization of complicated aneurysm anatomy as an adjunct to cerebral angiography.

## Methods

### Where to use the play dough models?

**Figure 1:**
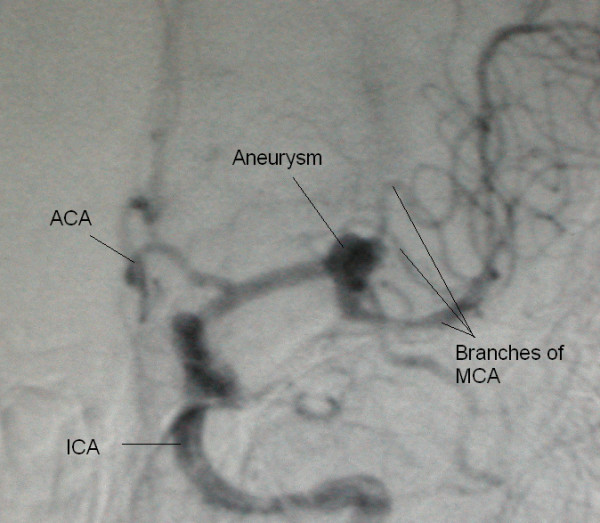
Middle cerebral artery aneurysm angiogram, anteroposterior view. ACA Anterior Cerebral Artery, ICA Internal Carotid Artery, MCA Middle Cerebral Artery. Aneurysm is seen in the trifurcation of MCA

**Figure 2:**
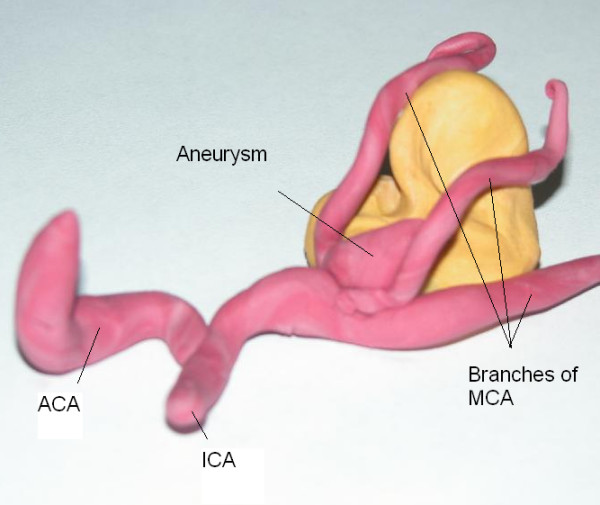
A Play Dough model based on the same patient's angiograms. ACA Anterior Cerebral Artery, ICA Internal Carotid Artery, MCA Middle Cerebral Artery. The model clarifies the location of the Aneurysm. Brain tissue shown in yellow.

We have used play dough as an adjunct to our traditional training tools and found some advantages for it. Its use is considered only in some visuospatially complicated aneurysms (figures 1 and 2) where imagination of the real anatomy based on 2D images or drawings may be difficult. It can be considered a good alternative for drawings sometimes done pre- and postoperatively by the consultants and residents in order to document what they are going to see or have seen during the operation. The preoperative model can be revisited after the operations to see where they went wrong.

### How to make the models?

The play dough used was not different from what children use for playing. Only two colors, red for vessels and yellow or white for nervous tissue have been used. The trainee studies the patient's angiograms and other available images and based on her or his previous experiences and knowledge, imagines the 3D structure of the lesion. Then using the play dough the trainee materializes her or his concept of the structure (figures 1 and 2). Since the goal is only to clarify the vascular anatomy, building of the models takes a short time (less than 5 minutes on average). There is no need for previous experience with play dough.

## Results

We have used this method for two years. In all cases, the preoperative models needed corrections and differed significantly from the true anatomy of the lesion. In those few cases where the models were made postoperatively, the results were much better, but interestingly the need for corrections in the minority of these cases could show us incorrect intraoperative 3D concept of the residents.

## Discussion

Since the number of neurosurgical residents is limited, we could not conduct an acceptable quantitative study regarding the comparison of play dough models with other teaching methods and report the advantages and disadvantages quantitatively.

### Advantages

Besides the low cost, availability and simplicity of use, play dough is much more efficient and realistic in training less experienced residents in comparison with the simple drawings and even angiographic views from different angles. It obviates the need for technological equipment. It helps discussion about the negative and positive points of different approaches with regard to the anatomy of surrounding vessels.

### Disadvantages

One of the major disadvantages of play dough is the psychological resistance of residents to the use in surgical training of something that usually is considered to be a toy. With time, this seems to lessen, especially when the constructed models help them present their questions or comments. It may be more acceptable to those trainees who have an artistic flair. Play dough is not as clean as drawings or computerized images. Use of computer software is not only more in vogue and acceptable, but also strengthens the computing skills of trainees.

Design and conduct of a study to compare the application of this tool to other training methods may help better evaluation of play dough as an educational tool.

## Conclusion

Although technologies like computerized software using the patients' own imaging data seems set to become more advanced in the future, use of play dough for some complicated cerebral aneurysm cases may be helpful in realistic 3D reconstruction of the lesions.

## Competing interests

The author(s) declare that they have no competing interests.

## Pre-publication history

The pre-publication history for this paper can be accessed here:


